# Correlation between percutaneous transthoracic needle biopsy and recurrence in stage I lung cancer: a systematic review and meta-analysis

**DOI:** 10.1186/s12890-020-01235-2

**Published:** 2020-07-20

**Authors:** Haichao Li, Rong Chen, Jian Zhao

**Affiliations:** Department of Thoracic Surgery, Qilu Hospital, Cheeloo College of Medicine, Shandong University, Jinan, 250012 Shandong China

**Keywords:** Percutaneous transthoracic needle biopsy, Stage I lung cancer, Recurrence, Meta-analysis

## Abstract

**Background:**

To systematically evaluate the correlation between percutaneous transthoracic needle biopsy (PTNB) and recurrence in stage I lung cancer.

**Methods:**

The databases of PubMed, EMbase, The Cochrane Library, CNKI, WanFang Data and China Biology Medicine disc were retrieved to collect relevant literatures about the correlation between PTNB and recurrence in stage I lung cancer. The retrieval time was limited from the time of their database establishment to April 30/2020.Screened the literature, extracted the data and assessed the quality of studies included. Then the meta-analysis was performed by using Stata l6.0 software.

**Results:**

A total of 8 cohort studies involving 2760 lung cancer patients were included. The results of meta-analysis showed that PTNB did not increase the risk of total recurrence and pleural recurrence in the patients with stage I lung cancer. The result of subgroup analysis is according to the tumor location. For stage I lung cancer, PTNB will increase the risk of pleural recurrence in patients with sub-pleural lesions but not in those without sub-pleural lesions.

**Conclusions:**

To stage I lung cancer, PTNB is not associated with the total recurrence and pleural recurrence but PTNB will increase the risk of pleural recurrence in patients with sub-pleural lesions.

Lung cancer is one of the malignant tumors with the highest morbidity and mortality in the world. In recent years, with the application of low-dose CT in screening for lung cancer, the rate of discovering lung mass has gradually increased. However, it is difficult to make qualitative diagnosis of lung occupying by imaging alone. Percutaneous transthoracic needle biopsy (PTNB) is more and more commonly used in the diagnosis of lung occupying lesions. But the operation’s safety of metastasis along the needle tract has not yet been determined. Some studies have shown that this operation will have the risk of breast wall implant metastasis [1], pleural metastasis [2, 3] and so on, while other studies showed that this operation is safe in the diagnosis [4, 5], and tumor metastasis along the needle track is relatively rare [6]. The purpose of this meta-analysis is to evaluate the risk of recurrence in patients with lung cancer caused by PTNB and to provide evidence-based medicine in clinical diagnosis.

## Materials and methods

### Literature inclusion and exclusion

#### Study type

Randomized controlled study and cohort study

#### Study subjects

Patients with stage I lung cancer

#### Intervention measures

Various types of PTNB, such as CT guided PTNB or ultrasound guided PTNB. Studies focusing on transbronchial or intraoperative needle biopsy will be excluded.

#### Exclusion criteria

① No PTNB study was conducted; ② letters, repeated publications, animal experiments, case reports, literature reviews, conference posters, conference abstracts and seminars; ③ four grid table values could not be obtained; ④ articles not published in English or Chinese and studies published in national journals of non-English speaking countries.

### Search strategies

Databases: PubMed, EMBASE, the Cochrane Library, CNKI, CBM and Wanfang database. Key words: “lung neoplasm”, “biopsy, needle”, “recurrence”, “randomized controlled trials” or “prospective studies” or “retrospective study”. The retrieval period is from the establishment of the database to 2020.04.30. The combination of subject words and free words is adopted in the retrieval, and there is no limit to the languages. At the same time, the references included in the research are supplemented by secondary retrieval. Taking PubMed as an example, the retrieval strategy is as follows:

1. ((((((((((((((((((“Lung Neoplasms”[Mesh]) OR Lung Neoplasms [Title/Abstract]) OR Pulmonary Neoplasms [Title/Abstract]) OR Neoplasms, Lung [Title/Abstract]) OR Lung Neoplasm [Title/Abstract]) OR Neoplasm, Lung [Title/Abstract]) OR Neoplasms, Pulmonary [Title/Abstract]) OR Neoplasm, Pulmonary [Title/Abstract]) OR Pulmonary Neoplasm [Title/Abstract]) OR Lung Cancer [Title/Abstract]) OR Cancer, Lung [Title/Abstract]) OR Cancers, Lung [Title/Abstract]) OR Lung Cancers [Title/Abstract]) OR Pulmonary Cancer [Title/Abstract]) OR Cancer, Pulmonary [Title/Abstract]) OR Cancers, Pulmonary [Title/Abstract]) OR Pulmonary Cancers [Title/Abstract]) OR Cancer of the Lung [Title/Abstract]) OR Cancer of Lung [Title/Abstract]
2. ((((((((((((“Biopsy, Needle”[Mesh]) OR Biopsy, Needle [Title/Abstract]) OR Biopsies, Needle [Title/Abstract]) OR Needle Biopsies [Title/Abstract]) OR Needle Biopsy [Title/Abstract]) OR Aspiration Biopsy [Title/Abstract]) OR Aspiration Biopsies [Title/Abstract]) OR Biopsies, Aspiration [Title/Abstract]) OR Biopsy, Aspiration [Title/Abstract]) OR Puncture Biopsy [Title/Abstract]) OR Biopsies, Puncture [Title/Abstract]) OR Biopsy, Puncture [Title/Abstract]) OR Puncture Biopsies [Title/Abstract]
3. ((((((“Recurrence”[Mesh]) OR Recurrence [Title/Abstract]) OR Recurrences [Title/Abstract]) OR Recrudescence [Title/Abstract]) OR Recrudescences [Title/Abstract]) OR Relapse [Title/Abstract]) OR Relapses [Title/Abstract]
4. (((((((((“Prospective Studies”[Mesh]) OR Prospective Studies [Title/Abstract]) OR Prospective Study [Title/Abstract]) OR Studies, Prospective [Title/Abstract]) OR Study, Prospective [Title/Abstract]) OR Prospective [Title/Abstract]) OR Prospectively [Title/Abstract])) OR (((((((“Retrospective Studies”[Mesh]) OR Retrospective Studies [Title/Abstract]) OR Studies, Retrospective [Title/Abstract]) OR Study, Retrospective [Title/Abstract]) OR Retrospective Study [Title/Abstract]) OR Retrospective [Title/Abstract]) OR Retrospectively [Title/Abstract])) OR (((((“Randomized Controlled Trial” [Publication Type]) OR Randomized Controlled Trial [Title/Abstract]) OR Randomized [Title/Abstract]) OR Randomizedly [Title/Abstract]) OR Placebo [Title/Abstract])
5. #1 AND #2 AND #3 AND #4

### Literature screenings and data extraction

The data included first author, publication time, sample characteristics (number, size, stage, metastasis, pleural recurrence, etc.) and so on. If there is a lack of relevant data in the literatures, contact the relevant authors, and if the relevant data cannot be obtained, they will be excluded.

### Quality evaluation

For randomized controlled trials, methodological quality was assessed using the five point Jadad scale. The bias risk of cohort studies were evaluated according to the Newcastle-Ottawa scale (NOS) and the results were cross-checked.

### Statistical analyses

We used Stata16.0 software for statistical analyses and the degree of heterogeneity was determined by the size of I^2^ value. I^2^ > 50% indicates obvious heterogeneity, we choose random effect model to merge statistics. I^2^ < 50% represents that there is small heterogeneity, we select the fixed effect model to merge statistics. For the comparison of the total recurrence rate and pleural metastasis rate of lung cancer between PTNB group and NPTNB group, the relative risk (RR) was used to represent the effect. The interval was estimated by 95% confidence interval (CI) and *p* < 0.05 means the difference was statistically significant.

## Results

### Literature screen

**Fig. 1:**
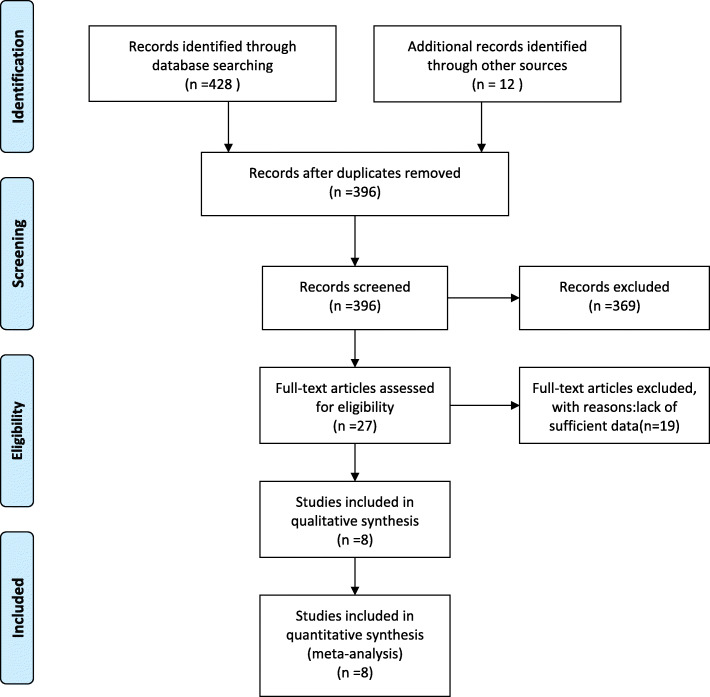
The literature screening process

We obtain 428 relative literatures, including 58 Chinese literatures and 370 English literatures. After reading the title and abstract, we excluded the literature that did not meet the inclusion criteria and included 10 articles. After reading the full text, we finally included 8 articles [2, 3, 7–12], with a total of 2760 patients having lung cancers. The literature screening process is shown in Fig. 1:

### Methodological quality evaluations of included studies

All included studies were cohort studies, assessed by the Newcastle-Ottawa 9-star scale (NOS). The NOS scale is an 8-point scoring tool used to evaluate the selection of study population, study comparability, follow-up results and study results. The score is used to evaluate the quality of literature research, with totaling 8 points. The evaluation results are shown in Table 1.

**Table 1:** Methodological quality evaluation of included studies

Inclusion study	Selection				comparabbility	Exposure			total
	adequate case definition	case representativeness	control selection	control definition		ascertainment of exposure	same method	non-response rate	
Matsuguma, H. 2005 [3]	1	1	1	1	1	1	1	1	8
Inoue, M. 2011 [2]	1	1	1	1	1	1	1	1	8
Asakura, K. 2012 [11]	1	1	1	1	1	1	1	1	8
Flechsig. P. 2015 [10]	1	1	1	1	0	1	1	1	7
Kashiwabara, K. 2016 [12]	1	1	1	1	1	1	1	1	8
Moon, S.M. 2017 [7]	1	1	1	1	1	1	1	1	8
Hu, C. 2018 [9]	1	1	1	1	0	1	1	1	7
Ahn, S. Y. 2019 [8]	1	1	1	1	1	1	1	1	8

### Basic characteristics of included studies

A total of 8 cohort studies were included. The basic characteristics of the included studies are shown in Table 2.

**Table 2:** Basic characteristics of included studies

Study object	Study type	Location	Sample size		Period	Median follow-up (months)		Stage	tumor size (mm)		Needle size	therapy
			PTNB	NPTNB		PTNB	NPTNB		PTNB	NPTNB		
Matsuguma, H. 2005 [3]	Cohort stud	Japan	66	224	1986,10- 2000.12	80	80	I	23.8	29.0/39.5	18 gauge	Surgery
Inoue, M. 2011 [2]	Cohort stud	Japan	131	316	1992–2008	66.5	57.9	I	25	27	18 gauge	Surgery
Asakura, K. 2012 [11]	Cohort stud	Japan	124	197	2002.10- 2009.02	45	42	I	19 ± 9	25 ± 9	18 gauge	Surgery
Flechsig. P. 2015 [10]	Cohort stud	Germany	26	9	2003–2010	17	17	I	Nr*	nr	15 gauge	Surgery chemotherapy, radiotherapy
Kashiwabara, K. 2016 [12]	Cohort stud	Japan	63	86	2009.04- 2014.03	43.2	43.2	I	21	27	21 gauge	Surgery
Moon, S.M. 2017 [7]	Cohort stud	Korea	243	149	2009.01- 2010.12	54	51	I	not sure	not sure	18/20 gauge	Surgery
Hu, C. 2018 [9]	Cohort stud	China	66	256	2010.01- 2014.09	not sure	not sure	I	25	23	18 gauge	Surgery,chemotherapy
Ahn, S. Y. 2019 [8]	Cohort stud	Korea	540	270	2004.01- 2010.12	63.7	64.1	I	26	20	17–22 gauge	Surgery

The total recurrence of lung cancer includes local recurrence and distant metastasis. Local recurrence is defined as any recurrence in the ipsilateral chest cavity including ipsilateral pleural recurrence. Distant metastasis refers to pleural, contralateral lung, extra-thoracic metastasis or recurrence of pericardial effusion. Pleural recurrence refers to the new development of new pleural nodules or pleural effusions detected by the chest CT scan. Pleural biopsy or pleural fluid cytology can confirm pleural recurrence.

#### The effect of PTNB on patients’ total recurrence rate

**Fig. 2:**
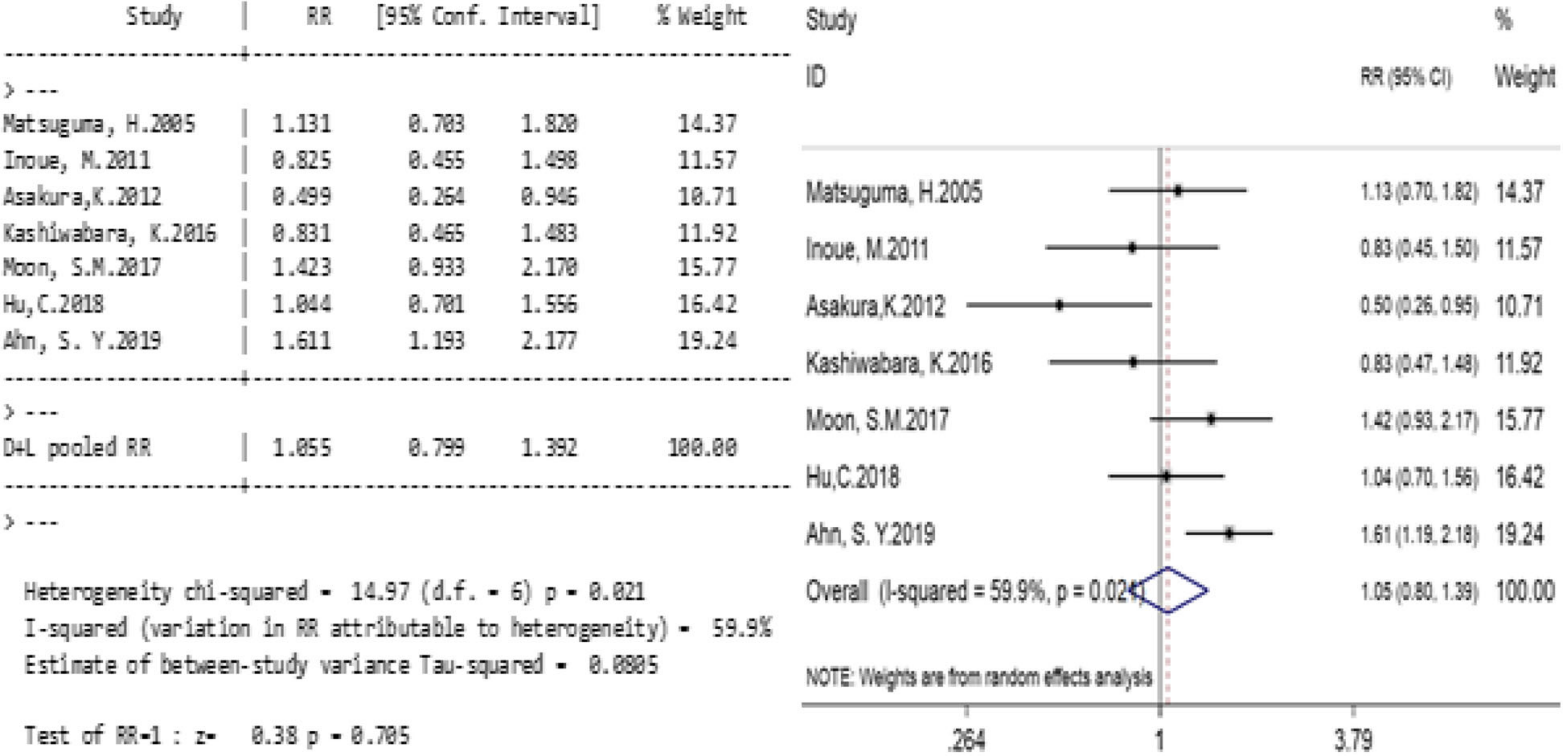
Relationships between PTNB and total recurrence rate. Legends: Significant heterogeneity was found in the study (I^2^ = 59.9%, *p* = 0.021). The total relative risk ratio (RR) was 1.055 (95% CI, 0.799–1.392; *p* = 0.705; Fig. 2)

Seven studies compared the total recurrence rate between the PTNB group and the non-PTNB group (Table 3). Significant heterogeneity was found in the study (I^2^ = 59.9%, *p* = 0.021; Fig. 2), so a random effect model was applied. The total relative risk ratio (RR) was 1.055 (95% CI, 0.799–1.392; *p* = 0.705; Fig. 2), indicating that there was no significant correlation between PTNB and the total recurrence rate of stage I lung cancers.

**Table 3:** Relationship between PTNB and total recurrence rate

Research object	PTNB		NPTNB		weight%	RR(95%CI)
	events	non-events	events	non-events		
Matsuguma, H. 2005 [3]	17	49	51	173	14.37	1.131 (0.703,1.820)
Inoue, M. 2011 [2]	13	118	38	278	11.57	0.825 (0.455,1.498)
Asakura, K. 2012 [11]	11	113	35	162	10.71	0.499 (0.264,0.946)
Kashiwabara, K. 2016 [12]	14	49	23	63	11.92	0.831 (0.465,1.483)
Moon, S.M. 2017 [7]	58	185	25	124	15.77	1.423 (0.933,2.170)
Hu, C. 2018 [9]	21	45	78	178	16.42	1.044 (0.701,1.556)
Ahn, S. Y. 2019 [8]	145	395	45	225	19.24	1.611 (1.193,2.177)
Total	279	1003	295	1203	100	1.055 (0.799,1.392)

#### The effect of PTNB on patients’ pleural recurrence rate

**Fig. 3:**
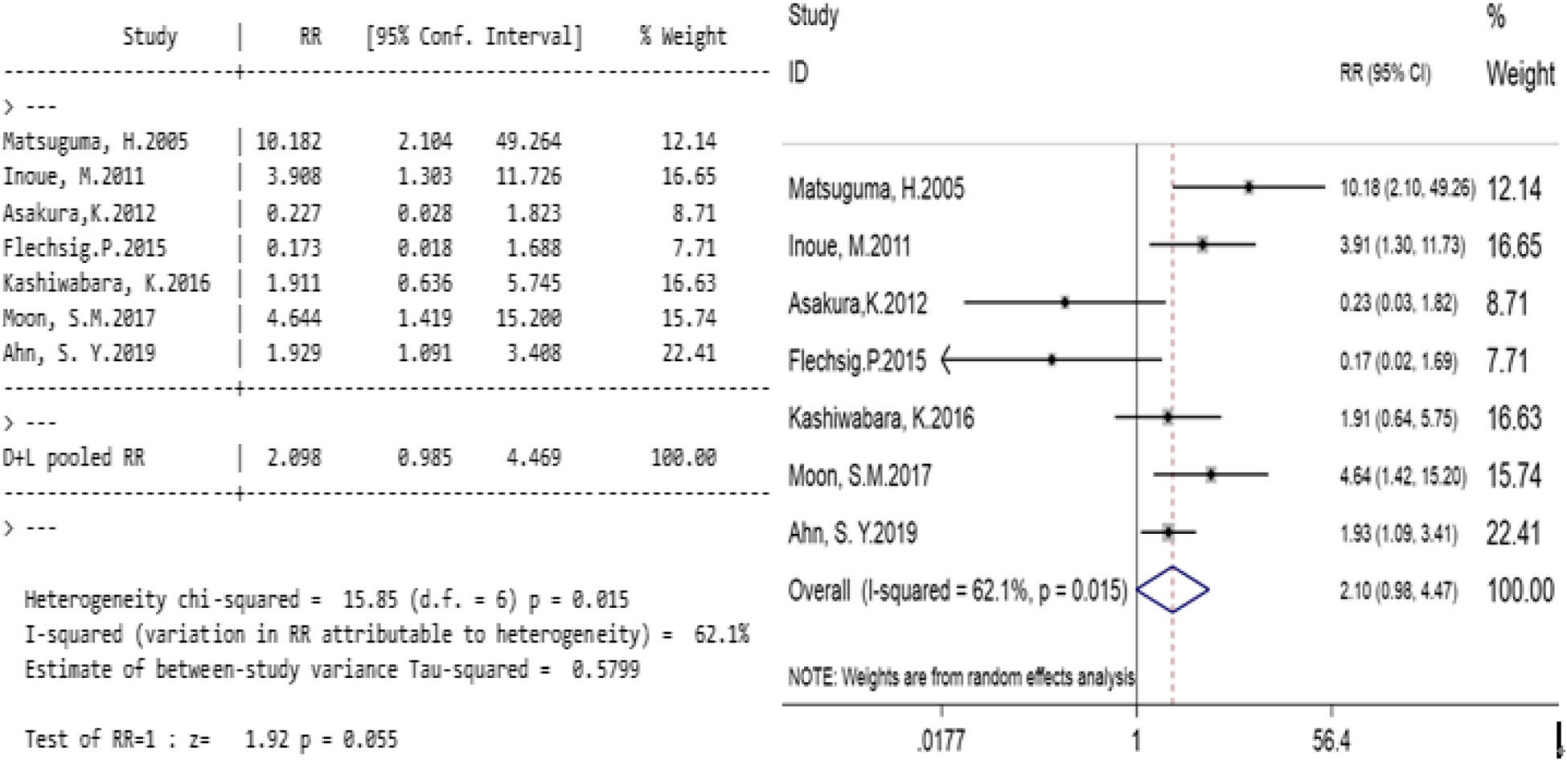
Relationships between PTNB and pleural recurrence rate. Legends: Significant heterogeneity was found in the study (I^2^ = 62.1%, *p* = 0.015; Fig. 3). The total relative risk ratio (RR) was 2.098 (95% CI, 0.985–4.469; *p* = 0.055; Fig. 3)

Seven studies compared the pleural recurrence rate between the PTNB group and the non-PTNB group (Table 4). Significant heterogeneity was found in the study (I^2^ = 62.1%, *p* = 0.015; Fig. 3), so using a random effect model. The total relative risk ratio (RR) was 2.098 (95% CI, 0.985–4.469; *p* = 0.055; Fig. 3), indicating that PTNB had no significant correlation with the pleural recurrence rate of stage I lung cancers.

**Table 4:** Relationship between PTNB and pleural recurrence rate

Research object	PTNB		NPTNB		weight%	RR(95%CI)
	events	non-events	events	non-events		
Matsuguma, H. 2005 [3]	6	60	2	222	12.14	10.182 (2.104,49.264)
Inoue, M. 2011 [2]	8	123	5	315	16.65	3.908 (1.303,11.726)
Asakura, K. 2012 [11]	1	123	7	190	8.71	0.227 (0.028,1.823)
Flechsig.P. 2015 [10]	1	25	2	7	7.71	0.173 (0.018,1.688)
Kashiwabara, K. 2016 [12]	7	56	5	81	16.63	1.911 (0.636,5.745)
Moon, S.M. 2017 [7]	23	223	3	146	15.74	4.644 (1.419,15.200)
Ahn, S. Y. 2019 [8]	54	486	14	256	22.41	1.929 (1.091,3.408)
Total	100	1096	38	1217	100	2.098 (0.985,4.469)

#### The effect of PTNB on the recurrence of pleura in patients with sub-pleural lesions

**Fig. 4:**
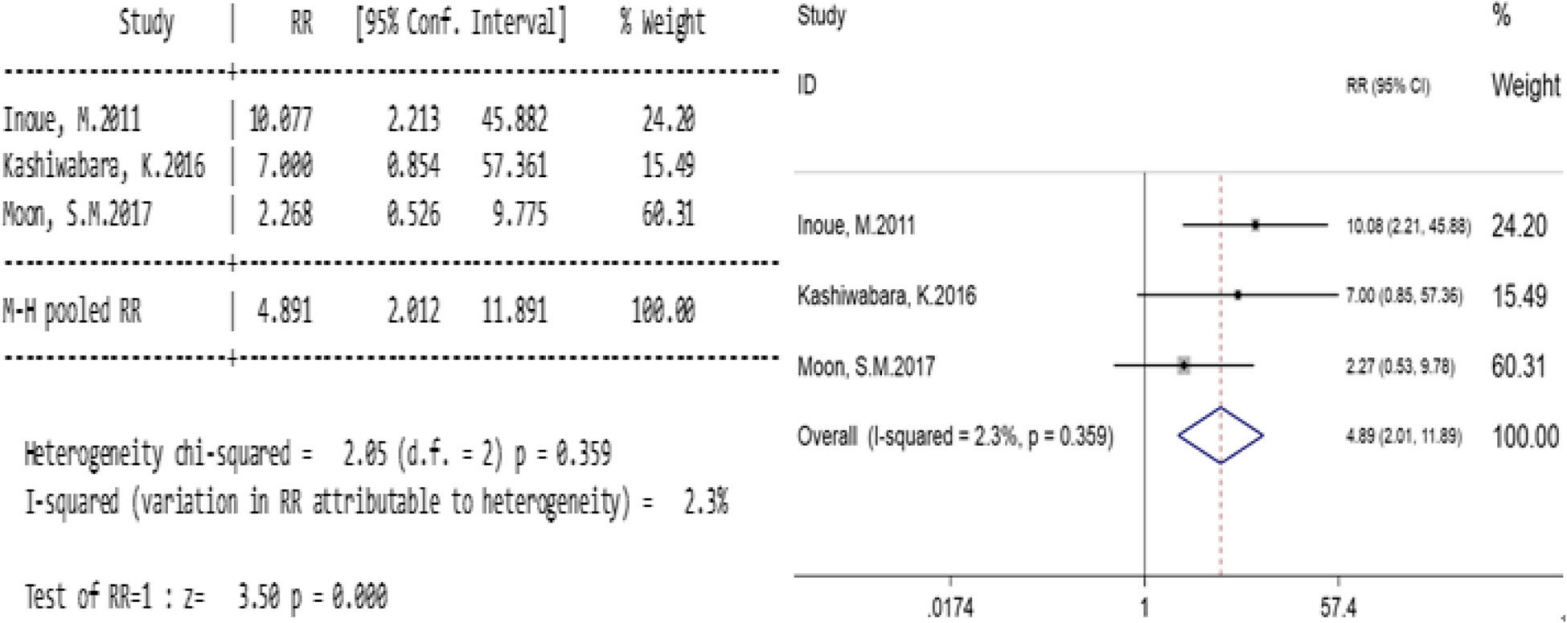
Relationship between PTNB and recurrence rate of sub-pleural lesions. Legends: A small heterogeneity was found in the study (I^2^ = 2.3%, *p* = 0.359; Fig. 4). For patients with sub-pleural lesions, the total relative risk ratio (RR) was 4.891 (95% confidence interval, 2.012–11.891; *p* = 0.000; Fig. 4)

Our study was analyzed by subgroup according to the tumor site. Three studies compared with pleural recurrence rates between PTNB and non-PTNB groups of patients with sub-pleural lesions (Table 5). A small heterogeneity was found in the study (I^2^ = 2.3%, *p* = 0.359; Fig. 4), and a fixed effect model was used. For patients with sub-pleural lesions, the total relative risk ratio (RR) was 4.891 (95% confidence interval, 2.012–11.891; *p* = 0.000; Fig. 4). The results indicate that for stage I lung cancers, PTNB will increase the pleural recurrence rate in patients with sub-pleural lesions.

**Table 5:** Relationship between PTNB and the pleural recurrence rate of the patients with sub-pleural lesions

Research object	PTNB		NPTNB		Weight%	RR(95%CI)
	events	non-events	events	non-events		
Inoue, M. 2011 [2]	8	44	2	129	24.2	10.077 (2.213,45.882)
Kashiwabara, K. 2016 [12]	4	12	1	27	15.49	7.000 (0.854,57.361)
Moon, S.M. 2017 [7]	11	86	2	38	60.31	2.268 (0.526,9.775)
Total	23	142	5	194	100	4.891 (2.012,11.891)

#### The effect of PTNB on pleural recurrence in patients without sub-pleural lung cancer

**Fig. 5:**
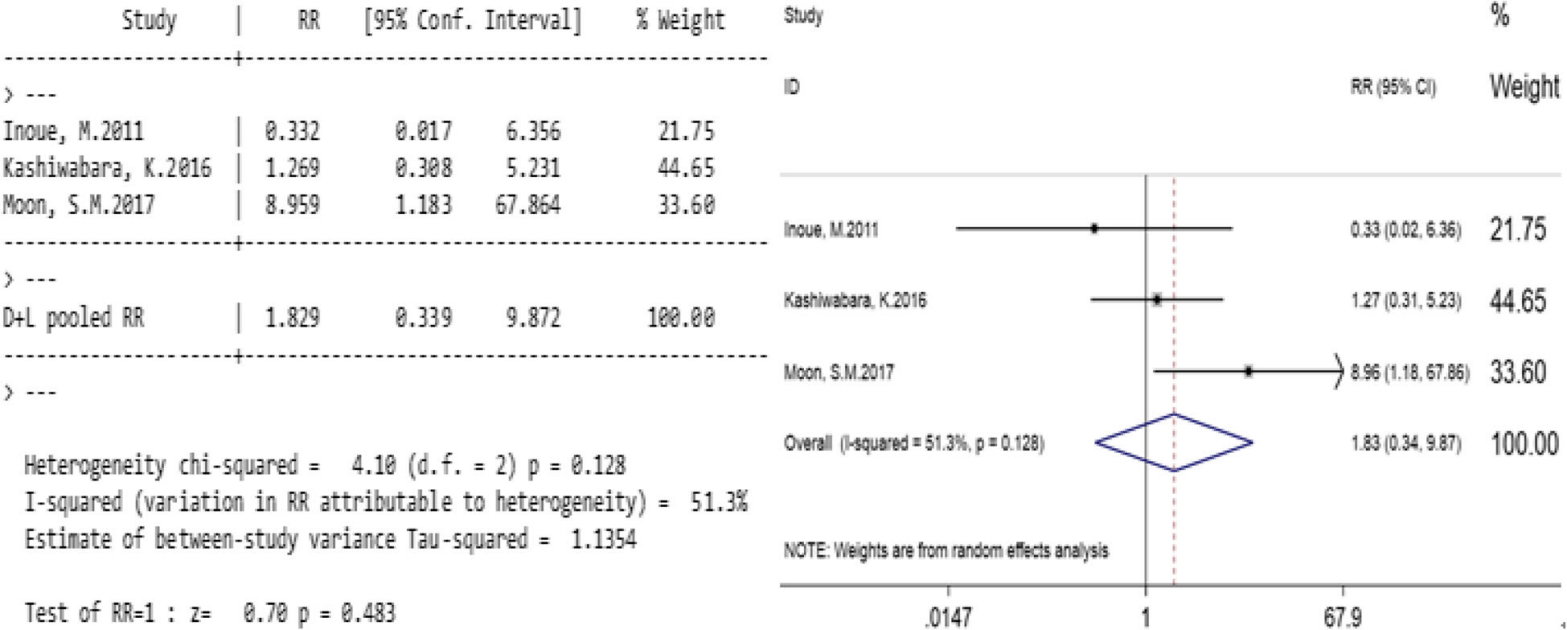
Relationship between PTNB and the pleural recurrence rate of the patients without sub-pleural lesions. Legends: Significant heterogeneity was found in the study (I2 = 51.3%, *p* = 0.128; Fig. 5). In patients without sub-pleural lesions, the total relative risk ratio (RR) was 1.827 (95% confidence interval, 0.339–9.872; *p* = 0.483; Fig. 5)

Three studies compared the pleural recurrence rate between PTNB and non-PTNB groups for patients without sub-pleural lung cancer (Table 6). Significant heterogeneity was found in the study (I2 = 51.3%, *p* = 0.128; Fig. 5), using a random effects model. In patients without sub-pleural lesions, the total relative risk ratio (RR) was 1.827 (95% confidence interval, 0.339–9.872; *p* = 0.483; Fig. 5). The results show that in stage I lung cancers, PTNB will not increase the risk of pleural recurrence in patients without sub-pleural lesions.

**Table 6:** Relationship between PTNB and the pleural recurrence rate of the patients without sub-pleural lesions

Research object	PTNB		NPTNB		Weight%	RR(95%CI)
	events	non-events	events	non-events		
Inoue, M. 2011 [2]	0	79	3	182	21.75	0.332 (0.017,6.356)
Kashiwabara, K. 2016 [12]	3	23	4	40	44.65	1.269 (0.308,5.231)
Moon, S.M. 2017 [7]	12	134	1	108	33.6	8.959 (1.183,67.864)
总计	15	236	8	330	100	1.829 (0.339,9.872)

#### The analysis of sensitivity

**Fig. 6:**
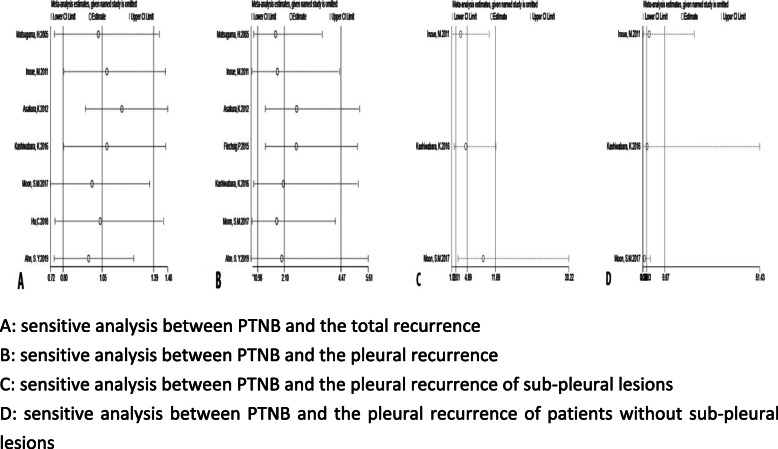
sensitive analyses. **a**: sensitive analysis between PTNB and the total recurrence. **b**: sensitive analysis between PTNB and the pleural recurrence. **c**: sensitive analysis between PTNB and the pleural recurrence of sub-pleural lesions. **d**: sensitive analysis between PTNB and the pleural recurrence of patients without sub-pleural lesions. Legends: For each analysis, the statistics are merged after each study is removed in turn, and the results do not change statistically

For each analysis, the statistics are merged after each study is removed in turn, and the results do not change statistically, indicating that the research results are reliable (Fig. 6).

#### Publication bias

The number of articles included is less 10, so no publication bias test was performed.

## Discussion

Tumors with different sizes and stages have different malignancy degree, so we limited the study to patients with stage I lung cancer. The main purpose of PTNB is to accurately identify the nodule’s quality for surgical resection, and to avoid unnecessary surgical treatment to patients with benign nodules. In recent years, PTNB has developed into one of the most commonly used diagnostic methods for diagnosis of lung lesions, especially for peripheral lung cancer. Although its diagnostic accuracy is high and there are few recent complications, it is still inevitable to destroy the lung structure during the process. Therefore, the puncture may spread tumor cells to the airways, blood vessels, pleural cavity, chest wall and so on, increasing the rate of lung cancer metastasis in potentially curable cancer, so it is still necessary to find the occurrence rate of long-term complications such as pleural recurrence. Since PTNB was first reported in 1965, some typical cases of pleural recurrence and needle channel implantation have been confirmed [1]. However, it was not until 2005 that Matsuguma and others conducted a cohort study and found that PTNB increased pleural recurrence in patients with stage I lung cancer [3]. After several studies conducted later, it was found that PTNB did not significantly increase the total recurrence rate of lung cancer. So it has not been agreed whether PTNB will increase the recurrence of pleura [2, 3, 7–12].

The results of this study did not find a significant correlation between PTNB and the total recurrence rate and pleural recurrence rate, indicating that the cancer recurrence rate is mainly related to the malignant degree of the primary tumor whereas not to the tissue destruction and tumor cells caused by lung cancer puncture. The tumor cells were separated from the original growth environment, so the probability of colonization and metastasis decreased in a new environment. In the included studies, most of the tumors in the PTNB group were larger than those in the NPTNB group and larger tumors were more likely to be accompanied by adverse results, so during comparison, they may mask the effect of PTNB on pleural metastasis and led to more false negative results. Therefore, there may be no significant correlation between PTNB and the recurrence rate, which should be further studied by improving tumor size homogeneity and eliminating related interference. But there are not enough typical samples at present.

It is worth noticing that compared with patients without sub-pleural lesions, PTNB increases the risk of pleural recurrence in stage I lung cancer patients with sub-pleural lesions. Because the location of the lesion is close to the pleura, some tumors are more likely to be accompanied by microscopic lymphoid infiltration, visceral pleural microinfiltration or pleural contact. These greatly increase the risk of pleural recurrence [7, 8], while the presence of visceral pleural invasion is not fully studied in their analysis. In the PTNB group, the pleural recurrence rate in patients with sub-pleural lesions (from 11 to 25%) was significantly higher than that in patients without sub-pleural lesions (from 0 to 12%). (Tables 5 and 6) While in the non-PTNB group, the pleural recurrence rate in the sub-pleural lesion group (2–5%) was similar to that in patients without sub-pleural lesion group (1–9%). (Tables 5 and 6).

Limitations: the main limitation is that the number of documents available is small and the sample size is insufficient. In addition, the included studies can not well balance the effects of many other confounding factors, such as tumor size, pathological differentiation and fibrosis state, puncture depth and times during the puncture, choice of puncture needle, intraoperative surgical methods, intraoperative use of chemotherapeutic drugs, postoperative follow-up time, postoperative adjuvant therapy and so on.

## Conclusions

To sum up, current evidence suggests that PTNB is not associated with an increase in the total recurrence rate and pleural recurrence rate in patients with stage I lung cancer. However, for patients with early sub-pleural lesions, PTNB will increase the risk of pleural recurrence, so it is still necessary to choose PTNB carefully. Because of the limitations of this study, large-scale, prospective and multicenter studies are still needed.

## Supplementary information

**Additional file 1.**

## Data Availability

The data supporting our findings can be found by contacting us (zhaojianjn@sdu.edu.cn).

## Abbreviations

PTNB: Percutaneous transthoracic needle biopsy
PRISMA: Preferred reporting items for systematic review and meta-analysis
SCLC: Small cell lung cancer
NSCLS: Non-small cell lung cancer
RR: Relative risk
